# The Search for Atmospheric Laminar Channels: Experimental Results and Method Dissemination

**DOI:** 10.3390/s22010158

**Published:** 2021-12-27

**Authors:** Iulian-Alin Roșu, Dragoș-Constantin Nica, Cătălin Dumitraș, Dragoș Chitariu, Luminița Bibire, Adrian Stelian Ghenadi, Valentin-Stelian Dragan, Maricel Agop

**Affiliations:** 1 Faculty of Physics, “Alexandru Ioan Cuza” University of Iasi, Bulevardul Carol I 11, 700506 Iasi, Romania; vastdragan@yahoo.com; 2 Department of Physics, “Gheorghe Asachi” Technical University of Iasi, 700050 Iasi, Romania; magop@tuiasi.ro; 3 Department of Geography, Faculty of Geography and Geology, “Alexandru Ioan Cuza” University of Iasi, Bulevardul Carol I 11, 700506 Iasi, Romania; 4 Faculty of Machine Manufacturing and Industrial Managements, “Gheorghe Asachi” Technical University of Iasi, 700050 Iasi, Romania; catalin.dumitras@tuiasi.ro (C.D.); chitariudragos@gmail.com (D.C.); 5 Department of Environmental Engineering and Mechanical Engineering, Faculty of Engineering, “Vasile Alecsandri” University of Bacău, 600115 Bacau, Romania; lbibire@gmail.com; 6 Department of Industrial Systems and Engineering, Faculty of Engineering, “Vasile Alecsandri” University of Bacău, 600115 Bacau, Romania; adrian_ghenadi@ub.ro; 7 Romanian Scientists Academy, 050094 Bucharest, Romania

**Keywords:** multifractal, laminarity, turbulence, atmosphere, lidar, algorithm

## Abstract

In this paper, a practical application of theoretical developments found in our previous works is explored in relation to atmospheric lidar data. Multifractal structures, previously named “laminar channels”, have been identified in atmospheric profiles—these exhibit cellular and self-structuring properties, and are spatially ordered across the atmospheric profile. Furthermore, these structures have been connected to the spontaneous emergence of turbulent behavior in the calm atmospheric flow. Calculating the location and occurrence of these channels can help identify features of atmospheric evolution, such as the development of the planetary boundary layer (PBL). Employing this theoretical background to atmospheric lidar data, attempts are made to confirm this suggestion and extract information about atmospheric structure and evolution by analyzing turbulent vortex scale dynamics and scale-corresponding Lyapunov exponents that form the basis of identifying the laminar channels in atmospheric lidar profiles. A parameter named “scale laminarity index” is then introduced, which quantifies the relation between vortex scale and chaoticity throughout the profile. Finally, the algorithmic methods employed in this study are described and distributed for future use.

## 1. Introduction

In general, the attempt to model atmospheric dynamics is performed with a combination of physical theories and computer-aided simulation [1,2,3,4,5]. The theoretical aspect of these models can be difficult and obtuse, since the chaotic conditions describing most natural fluid flows make it especially difficult to find universality classes, however, since even computational simulations employ specific algorithms found through theoretical efforts, such developments are important [4,5,6,7]. It is then possible to divide two types of such theories, both of which are based on conservation laws: differentiable on integer dimensions, and non-differentiable for fractal and multifractal entities [1,2,3,4,5,6,7].

Recently, a new class of models to describe atmosphere dynamics based on scale relativity theory, either employing monofractal dynamics or using the multifractal dynamics as in the case of the multifractal theory of motion, has been developed [8,9,10]. Thus, by presupposing that the atmosphere contains, and is consisted of, multifractal objects, then the dynamics of these objects can be described through their motions which are dependent on scale resolution on continuous and non-differentiable multifractal curves. As discussed in this introduction, notions such as scale transition and symmetry breaking and invariance are necessary in order to understand atmospheric dynamics if multifractality is considered. Because of this, a scale transition relation shall be used to exemplify gauge construction, and shortly explain the scaling model employed in this study.

Here, we consider the sequence of the values of the variable x, wherein a multifractal function F(x) with x∈[a,b] can be associated with an atmospheric variable:

(1) $$x_{a}=x_{0},\;x_{1}=x_{0}+\varepsilon ,\ldots ,\;x_{k}=x_{0}+k\varepsilon ,\;\ldots ,\;x_{n}=x_{0}+n\varepsilon =x_{b}$$

We can denote with F(x,ε) the broken line connecting the following points:

(2) $$F\left(x_{0}\right),\;\ldots ,\;F\left(x_{k}\right),\;\ldots ,\;F\left(x_{n}\right)$$

Thus, let F(x,ε) be an ε-approximation scale. Next, it has been shown that by considering that for an arbitrary but fixed $\varepsilon_{0}$:

(3) $$F\left(x,\varepsilon^{\prime }\right)=\left[1+\frac{\partial }{\partial \ln\left(\frac{\varepsilon }{\varepsilon_{0}}\right)}\frac{d\varepsilon }{\varepsilon }\right]F\left(x,\varepsilon \right)$$

where $\varepsilon^{\prime }=\varepsilon +d\varepsilon$ represents an infinitesimal scale transformation. The following equation:

(4) $$\hat{D}=\frac{\partial }{\partial \ln\left(\frac{\varepsilon }{\varepsilon_{0}}\right)}$$

is a dilation or contraction operator, and thus the intrinsic resolution variable is $\ln\left(\frac{\varepsilon }{\varepsilon_{0}}\right)$. itself [11]. With this, the spontaneous breaking of scale invariance of a given multifractal variable describing atmospheric dynamics implies:

(5) $$\frac{\partial F}{\partial \ln\left(\frac{\varepsilon }{\varepsilon_{0}}\right)}=P\left(F\right)$$

where P(F). is a function for which the following is true:

(6) P(F)≠F

By picking the second-simplest possible gauge, P(F)=BF, in which B is an arbitrary coefficient, we obtain:

(7) $$\frac{F}{F_{0}}={\left(\frac{\varepsilon }{\varepsilon_{0}}\right)}^{B}$$

In this manner, the spontaneous breaking of scale invariance can become the mechanism through which the turbulent energy cascade operates from large to small scales.

These theories are important because they represent foundations to practical applications of atmospheric modeling, with applications in various climate studies, meteorology, and atmospheric aerosol transport. Indeed, the set of theories presented in this work leads to a novel application of lidar data which can be used to estimate vertical atmospheric dynamics, which shall be shown in a later segment. Furthermore, they are important even on a purely theoretical basis, as potential avenues into offering a complete understanding of the mathematical apparatus, which describes atmospheric turbulence.

## 2. On Turbulent Scale Evolution through Gauges and Equivalences with the Logistic Map

In general, whatever scaling gauge is employed, energy is injected through vortices of length $L_{0}$, which triggers a “cascade” of intermediary $l_{n}$ vortices towards minimal vortices of dissipation scale $l_{d}$. The scale evolution found from the previously described gauge through the series of discrete length scales is:

(8) $$l_{n}\left(n\right)=L_{0}2^{-n},\;n=0,1,2,\ldots$$

The common β-constant model starts with the assumption that energy dissipation can be distributed homogenously throughout uniformly dimensional fractal eddies; this assumption is also named “absolute curdling” [12]. In fact, this model is produced by presupposing $\frac{\varepsilon }{\varepsilon_{0}}=2$, B=−n, and F=l. Recently, however, we have expanded upon this model in previous studies and successfully implemented it in a practical framework through lidar data [11,13]. It has been found that this model belongs to a class of gauges obtained from scale transition laws in the context of multifractality and that a similarly simple, yet slightly different, model containing a scale resolution parameter can give improved results [13]. By maintaining a constant β, but by adding the scale resolution parameter, the following equation:

(9) $$l_{n}\left(n,s\right)=L_{0}2^{-ns^{2}}$$

leads to multiple vortices that have their own differing fractal dimensions, i.e., leads to multifractal flow [13]. Because of the scale resolution dependency, s. shows how rapidly the scales of the turbulent cascade decrease in size. Multiple other parameters pertaining to turbulent flow can then be calculated through this model, and these results have been previously published [11,13]. Furthermore, this theory has been applied before to lidar data, obtaining time series of these various parameters, and it has been found that these results are in accord with existing scientific literature, especially with regards to the known behavior of the PBL [11,13].

Next, this model can be connected to a modified form of the logistic map, which approximates under certain conditions the behavior of a multifractal velocity mode of atmospheric flow. Usage of this model in the logistic map implies an associated Lyapunov exponent for each stage of the turbulent flow—in this manner, it is possible to determine whether for a given set of initial parameters and at a given altitude in an atmospheric column there exist stages in the turbulent cascade that present momentary laminar qualities. The manner in which the connection to the logistic map is done shall be shortly detailed in the following segment: following a Galerkin projection of multifractal type of the nondimensional Navier-Stokes equations of multifractal type, it is possible to write an arbitrary velocity Fourier mode of these equations with the iterative equation:

(10) $$a_{n+1}=4\left(1-Re^{-1}\tau {\left|k\right|}^{2}\right)a_{n}\left(1-a_{n}\right)=ra_{n}\left(1-a_{n}\right)$$

which is equivalent to the logistic map, where Re is Reynold’s number, τ is a time step parameter, and k. is a wavenumber associated with the velocity field decomposition [13]. Other studies have also been written which prove equivalence between the logistic map and the Navier-Stokes equations [14,15]. It is obvious that because the fact that r depends on the time scale and wavenumber, these factors need to be less than Re. so that the condition r>0 to hold and for the equation to be valid. In the following segment, the parameters are identified. It is understood what Reynold’s number represents; the following popular approximation is used to determine it:

(11) $$Re={\left(\frac{L_{0}}{l_{d}}\right)}^{\frac{4}{3}}$$

After this, the interpretation of the arbitrary wave vector k is debatable, however, the standard interpretation $\left|k\right|=\frac{1}{l_{n}}$ may be applied. However, some theoretical considerations could provide a different interpretation. The equation describing $l_{n}$ produces length scales that decrease in size from supra-unitary to sub-unitary measures of length—if the standard interpretation mentioned before is used, r will evolve from 4 to 1 if τ=ct., which is exactly the opposite of what the model should produce. Given the typical bifurcation sequence for the Navier-Stokes equations, which is steady→periodic→quasiperiodic→chaotic, it is unrealistic for the turbulent flow to be modelled in a way that corresponds r=4 to the largest scales because this would be precisely opposite to the typical bifurcation sequence [16]. Of course, the previously mentioned sequence, which is the “standard route to chaos”, is followed by the logistic map. The elements in r must decrease, instead of increase, because $Re^{-1}\tau {\left|k\right|}^{2}$ shall end up decreasing in time, and $4\left(1-Re^{-1}\tau {\left|k\right|}^{2}\right)$ will increase. It is possible to fix this by considering τ a variable decreasing quantity, $\tau_{n}$, however, it is unclear what physical significance one could ascribe to this quantity given the fact that it is a time step parameter introduced through a simple forward Euler single-step explicit time integration procedure.

Now, if the differential equation used to yield Equation (10) is solved, the result is a Bernoulli equation solution that describes the velocity mode a(t); however, it is not helpful in this context. Then, returning to the timestep parameter, if it is considered variable, as previously suggested, then one would have to assume that $4\left(1-\frac{\tau_{0}}{ReL_{0}{}^{2}}\right)=1$, because 1 is the transcritical point of the logistic map where the modeled turbulent flow is supposed to begin. Then, this implies that the time step of the velocity mode of the initial turbulent vortex is $\tau_{0}=\frac{3ReL_{0}{}^{2}}{4}$. However, when considering that in atmospheric flows $L_{0}$ values on the order of hundreds of meters and $l_{d}$ on the order of millimeters, or at most centimeters, are common, then $\frac{1}{ReL_{0}{}^{2}}$ will always be on orders of magnitude between $10^{-10}$ and $10^{-12}$. Finally, this results in the fact that the time step parameter of the initial turbulent vortex should present values of the order $10^{10}-10^{12}s$, which are equivalent to hundreds, even thousands, of years, which is obviously not physically realistic. Perhaps the most efficient and simple solution, and the least computationally intensive one, is to employ the following equation:

(12) $$a_{i+1}=4\left[1-l_{n}{}^{2}\left(\frac{l_{d}{}^{\frac{7}{3}}}{u_{d}L_{0}{}^{\frac{4}{3}}}\right)\right]a_{i}\left(1-a_{i}\right)$$

which implies an r with good, predictable results that conforms to the behavior of the standard logistic map. Here the time step has been considered constant, in terms of the minimal length scale and minimal velocity as $\tau \equiv t_{d}=\frac{l_{d}}{u_{d}}$. $u_{d}$ itself can be modeled through a multifractal vortex procedure that requires the minimal turbulent scale [11].

Considering the connection between the logistic map and atmospheric turbulence scale models, the approach is quite novel. However, even regarding the connection between the logistic map and atmospheric turbulence studies, not much has been written; Frisch has discovered that a mathematical expression similar to the logistic map might be considered “a poor man’s Navier-Stokes equation” [15]. A few other studies have also explored the connection between the logistic map and equations that describe two-dimensional turbulence [14,17].

In any case, after this connection to lidar data, scaling models and the logistic map has been performed, it is then possible to approximate whether or not certain stages of atmospheric turbulence, at a given altitude and time, present quasi-laminar behavior. Turbulent or laminar flow regimes can be identified in this manner throughout the atmosphere, because for certain initial values, and at certain scales and periods, it is then possible to arrive at such r values that characterize laminar regions of the logistic map, which would imply that, at certain scales and altitudes, atmospheric flow can spontaneously exhibit laminar or quasi-laminar behavior. This can then be used to construct a new measure of turbulence or lack thereof, named the “scale laminarity index”:

(13) $$S_{\lambda }=\langle \lambda_{n}l_{n}\rangle$$

which is composed of the averaging of all scales and the Lyapunov indices given by the r values produced by the scales at a given altitude. It stands to reason that if $S_{\lambda }$ is large then the number of turbulent stages is large and positive Lyapunov exponents are associated with large scales, which indicates large scale fully developed turbulence. So far, according to the presented theory, it is clearly suggested that laminar-chaotic and vice-versa transitions might occur spontaneously, given the correspondence between the logistic map and the Navier-Stokes equation and the sudden “islands of stability” found in the logistic map. This shows that laminar channels exist and propagate throughout the atmosphere, and they do so in a cellular, self-structuring manner according to equations developed from the multifractal theory of atmospheric motion. In fact, such structures have already been found to exist through experimental lidar data, and in the following segment of the paper, their existence and characteristics can be analyzed again [13,18].

## 3. Processing and Analysis of Experimental Data

Experimental data obtained via a lidar platform are introduced in order to apply the theory written so far and verify the results. The method used to process these data is described in greater detail in our past works, however, they rely on obtaining the structure coefficient of the refraction index profile $C_{N}^{2\;}$ in the following manner [11,19]:

(14) $$\;\sigma_{I}^{2}\left(L\right)=1.23\;C_{N}^{2\;}\left(L\right)k_{\lambda }{}^{\frac{7}{6}}L^{\frac{11}{6}}$$

with $\sigma_{I}^{2}$ being the “scintillation” or the logarithm of the standard deviation of light intensity of a source of light observed from a distance represented by the optical path L. The following definition can be given:

(15) $$\sigma_{I}^{2}\left(L\right)=\ln\left(1+\frac{\langle I{\left(L\right)}^{2}\rangle -\langle I{\left(L\right)}^{2}\rangle }{\langle I{\left(L\right)}^{2}\rangle }\right)$$

where I is the intensity of the backscattered range-corrected lidar signal at the particular point in the optical path, i.e., the RCS (Range Corrected Signal) intensity. Usually, in past studies we have considered it sufficient to employ three RCS profiles in the averaging process. After the $C_{N}^{2\;}$ profile has been determined, it is now possible to calculate, with a degree of approximation, the length scales. The inner scale profile is linked to scintillation:

(16) $$\sigma_{I}^{2}\left(L\right)≅0.615\;C_{N}^{2\;}\left(L\right)L^{3}l_{d}{\left(L\right)}^{\frac{-7}{3}}$$

and the outer scale is linked to the $C_{N}^{2\;}$ profile:

(17) $$C_{N}^{2}\left(z\right)=L_{0}{\left(z\right)}^{\frac{4}{3}}{\left(\nabla \langle n\left(z\right)\rangle \right)}^{2}$$

In the case of turbulent eddies that are situated in the inertial subrange, it is possible to approximate the refraction index profile from the definition of the structure coefficient:

(18) $$n\left(z\right)≅n_{0}-\sqrt{C_{N}^{2}\left(z\right)z^{\frac{2}{3}}}.\;$$

which can then be used to extract the outer scale profile. This method is well-referenced in our studies and has been already used successfully multiple times.

Now, once the outer and inner/dissipation scales have been obtained, it becomes feasible to use the modified beta model, and verify the hypotheses established in this work, however, given the previous presentation of the theories employed in this study, a final summary is required. We consider that atmospheric dynamics can be described by nondifferentiable yet continuous multifractal entities that evolve and bifurcate according to certain gauges. Then, these gauges can be used to construct scaling models that describe the evolution of these entities, and such models can be connected to the logistic map, given the fact that the logistic map itself can represent a velocity mode of a multifractal Navier-Stokes equation. With this connection in place, each scale becomes associated with a Lyapunov exponent, thus making it possible to determine whether or not certain scales at certain altitude exhibit chaotic or laminar channels—it will also be seen that laminarity does occur and that it occurs in an ordered manner, as channel-like structures.

Remote sensing data can then be used to further apply the theory discussed thus far. Numerous studies have been performed strictly regarding environmental observation through remote sensing or other remote-controlled platforms; manned low Earth orbit platforms (MLEOPs) provide laboratories for scientific experiments in a wide range of disciplines, some of them even presenting lidar technology, such as the Lidar In-space Technology Experiment (LITE) developed by NASA [20]. On the other hand, with its high spatial resolution and low cost, Unmanned Aerial Vehicles (UAVs) have become a promising tool for monitoring various environmental aspects [21]. In this case, however, elastic lidar data shall be used to validate our theory.

The RCS data used in this study have been obtained from the RALI Multiwavelength Raman Lidar Platform, which is a part of the National Institute for Research and Development in Optoelectronics INOE 2000 in Bucharest, Romania. The laser emission wavelengths are 1064 nm (90 mJ), 532 nm (50 mJ) and 355 nm (60 mJ) and the detection channels are 1064, 532 cross, 532 parallel, 355 nm (elastic wavelengths), and 607, 387 and 408 nm (Raman channels). The laser pulse duration is 7–9 ns, repetition rate 10 Hz, and the beam diameter between 5.5–7 mm at FWHM (Full Width at Half Maximum). The dynamic range covers 2–15 km depending on atmosphere transmission, with a 3.75 m spatial resolution. The reception has a 400 mm Cassegrain telescope with 1.73 mrad field of view, and the system acquisition is both analog and photon counting, with a 20 MS/s analog sampling rate and 250 MHz photon-counting count rate. The RALI lidar system was upgraded and tested against other lidar systems, and its results have been published in multiple other studies [22,23,24].

An analysis of the chaotic behavior of the turbulent cascade in an atmospheric profile is then performed in the following manner: by obtaining $l_{n}$ at given altitudes throughout the possible stages in the profile, several r values are obtained which are then used to calculate corresponding maximal Lyapunov exponents. These exponents can then be used in order to quantify the chaoticity of the given stage of the turbulent cascade at a given altitude. It should be observed that a higher number of stages is reached near areas of stability of the profile, and our recent studies indicate that such areas might show the altitude of the PBL [11,13]. A careful examination of the profiles will reveal what appear to be ascending or descending structures which have been previously named “laminar channels”, and their presence is a consequence of the fact that, at certain altitudes, the r parameter calculated through the scales obtained by lidar data corresponds to a region of the logistic map that is characterized by a negative Lyapunov exponent, and then, at different altitudes and scales, this correspondence repeats itself. Thus, it is possible to identify “ascending” or “descending” laminar channels, which can help explain atmospheric transport phenomena; horizontal channels of indeterminate effect and competing channels of both ascending and descending variety can be found. Areas where channels are horizontal or competing should indicate stable sections of the profile, where atmospheric structures at that given scale and altitude neither decrease nor increase in altitude. It is worth mentioning that an absence of such laminar channels indicates areas of high turbulent activity. Regarding negative values and the representations in our plots, the value 0 has been manually set as the inferior limit of the colormap in order to prevent any representation ambiguity regarding which segments are laminar.

**Figure 1:**
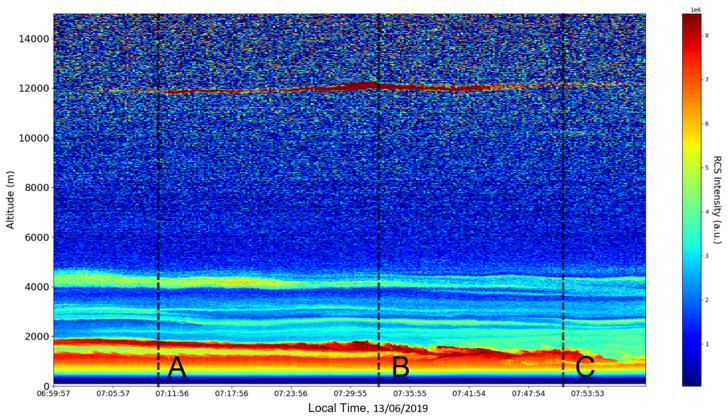
RCS timeseries, Bucharest, Romania, 13 June 2019.

**Figure 2:**
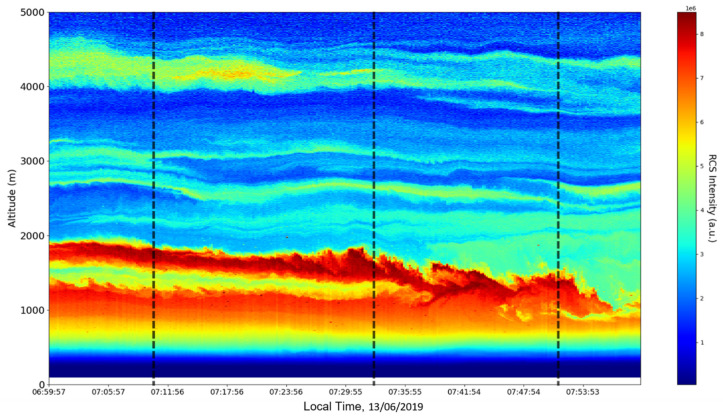
RCS timeseries, Bucharest, Romania, 13 June 2019 (zoomed-in, inferior region of interest).

**Figure 3:**
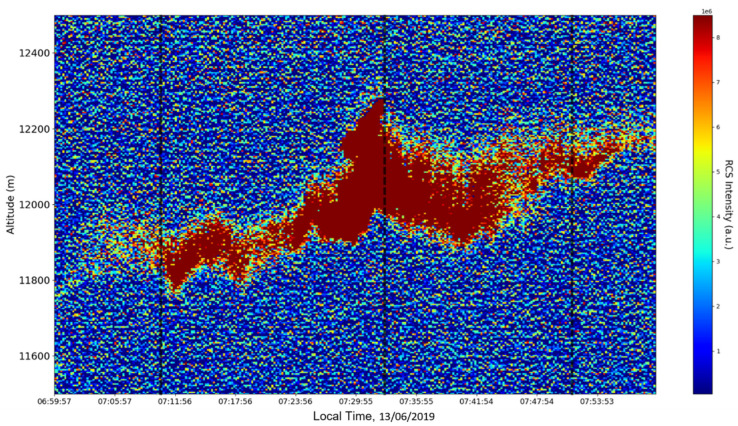
RCS time-series, Bucharest, Romania, 13 June 2019; zoomed-in, superior region of interest.

Now, in our previous studies, we have hypothesized that, because the turbulent cascade proceeds towards the dissipation scale, ascending channels represent upward transport and descending channels show downward transport. Thus, it stands to reason that a preliminary analysis should be made using the plots in order to confirm any such correlations. The first set of figures shows a time series plot of lidar profiles taken approximately from 07:00:00 PM UTC to 08:00:00 PM UTC with the previously mentioned lidar platform (Figure 1, Figure 2 and Figure 3). The chosen emission wavelength channel is 1064 nm. Various features of the atmosphere can be directly observed, including but not limited to stratospheric cirrus clouds, pollutant plumes and the PBL (Figure 1, Figure 2 and Figure 3).

The first zoomed-in figure of the time series shows multiple pollutant plumes and other structures associated with the PBL—it possible to confidently exclude the presence of clouds in the lower atmosphere, given the fact that Meteomanz.com displays clear conditions for the “BUCURESTI FILARET” station which is closest to the lidar platform, and also given the fact that ACTRIS lidar data collecting procedure demands that data collection start only during clear conditions (Figure 2). Meanwhile, the third zoomed-in figure of the time series exhibits what appears to be a very high stratospheric cirrus-type cloud (Figure 3).

**Figure 4:**
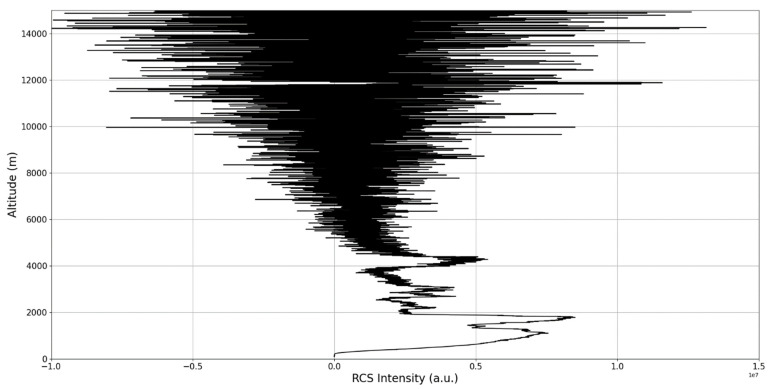
RCS profile corresponding to point A.

**Figure 5:**
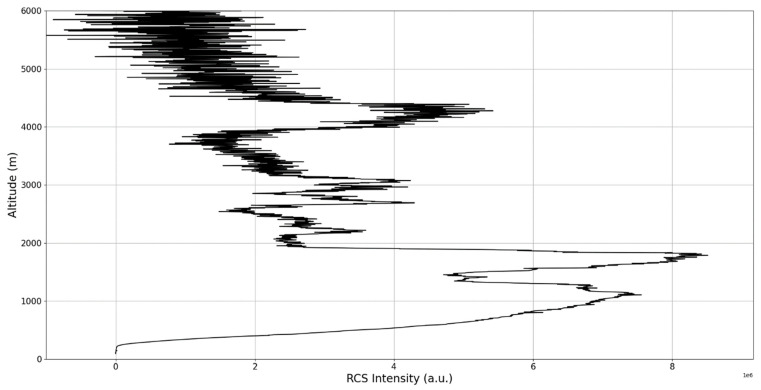
Zoomed-in RCS profile corresponding to point A.

**Figure 6:**
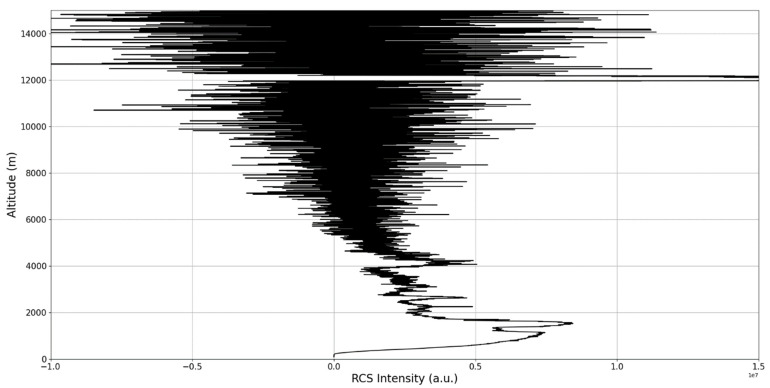
RCS profile corresponding to point B.

**Figure 7:**
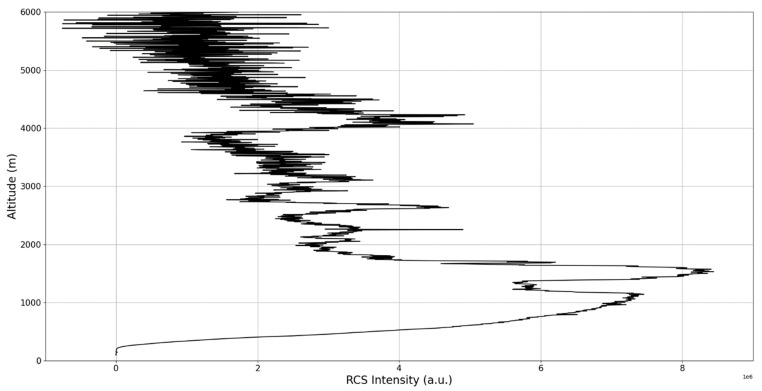
Zoomed-in RCS profile corresponding to point B.

**Figure 8:**
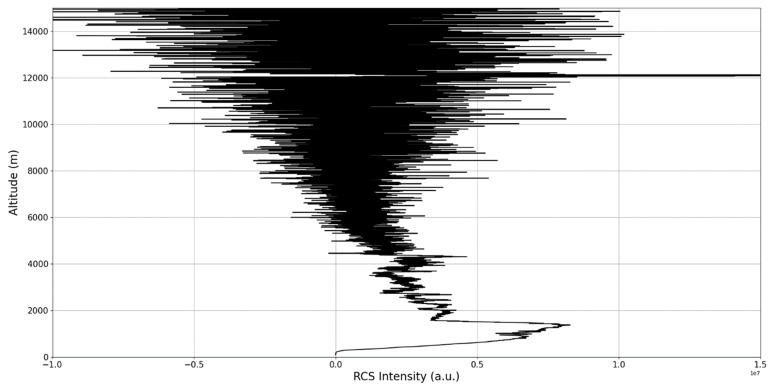
RCS profile corresponding to point C.

**Figure 9:**
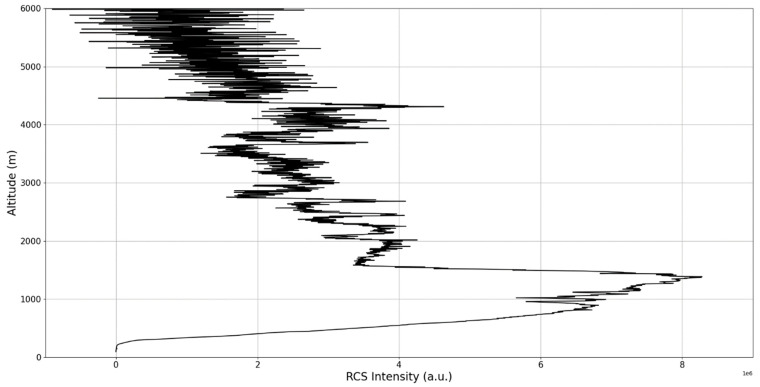
Zoomed-in RCS profile corresponding to point C.

After the visual analysis of this RCS dataset, three significant points in the time series, namely 07:10:00, 07:33:00 and 07:51:00, are chosen, which shall be named “point A”, “point B”, and “point C”, respectively. These three points seem to be the initial times of certain atmospheric evolutions that are most visible, and the location of these evolutions and trends shall be arbitrarily determined (Figure 1, Figure 2, Figure 3, Figure 4, Figure 5, Figure 6, Figure 7, Figure 8 and Figure 9). The position and profiles associated with these three points have been delineated consecutively with three dashed black lines.

**Figure 10:**
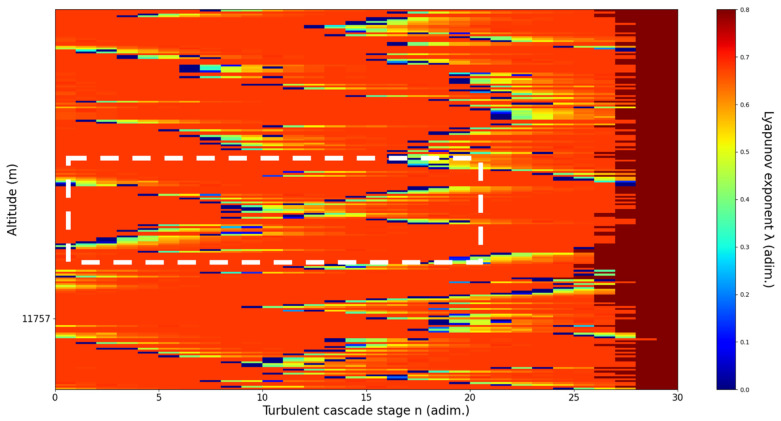
Lyapunov exponent per turbulent cascade stage colormap altitude plot, Bucharest, Romania, 13 June 2019, 07:10:00; point of interest: 11,900 m.

**Figure 11:**
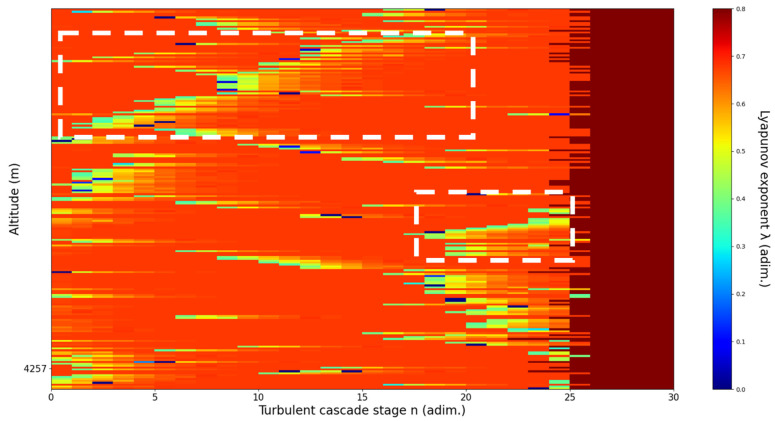
Lyapunov exponent per turbulent cascade stage colormap altitude plot, Bucharest, Romania, 13 June 2019, 07:10:00; point of interest: 4500 m.

**Figure 12:**
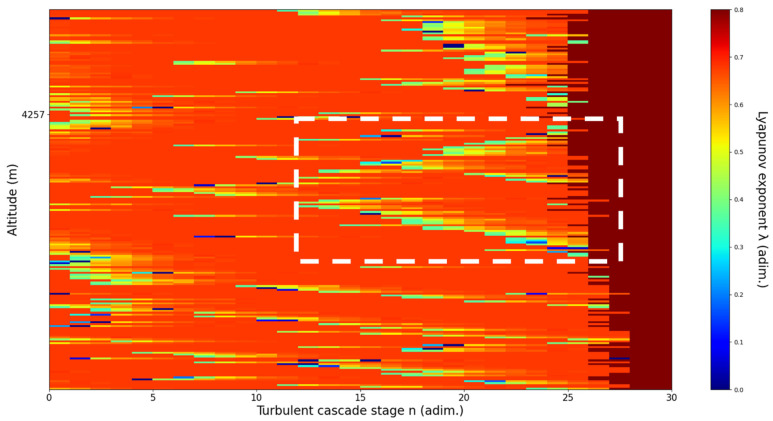
Lyapunov exponent per turbulent cascade stage colormap altitude plot, Bucharest, Romania, 13 June 2019, 07:10:00; point of interest: 4000 m.

**Figure 13:**
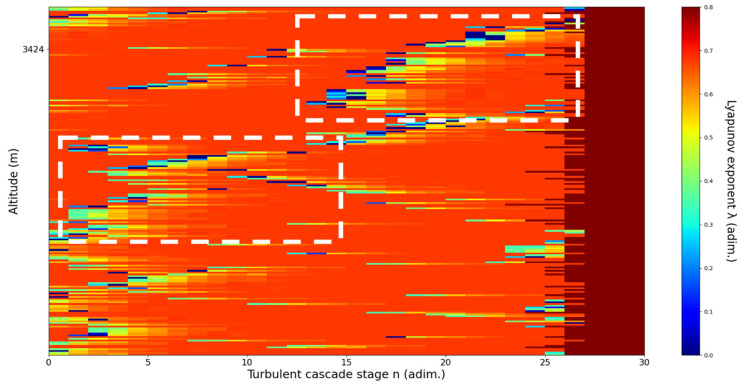
Lyapunov exponent per turbulent cascade stage colormap altitude plot, Bucharest, Romania, 13 June 2019, 07:10:00; point of interest: 3051 m.

**Figure 14:**
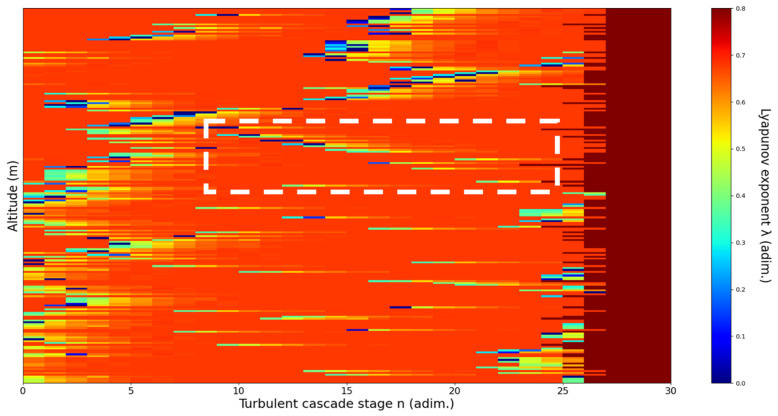
Lyapunov exponent per turbulent cascade stage colormap altitude plot, Bucharest, Romania, 13 June 2019, 07:10:00; point of interest: 2939 m.

**Figure 15:**
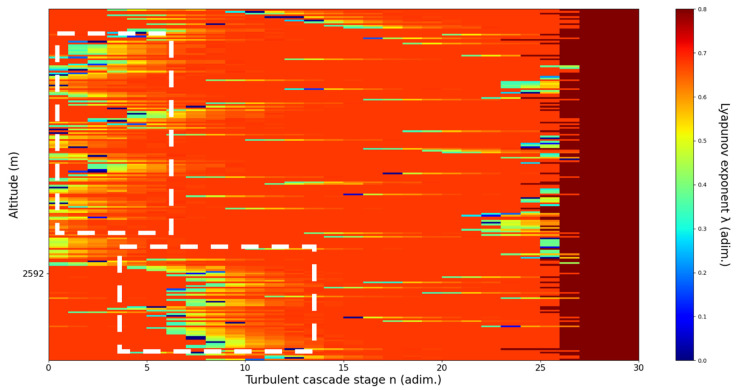
Lyapunov exponent per turbulent cascade stage colormap altitude plot, Bucharest, Romania, 13 June 2019, 07:10:00; point of interest: 2689 m.

**Figure 16:**
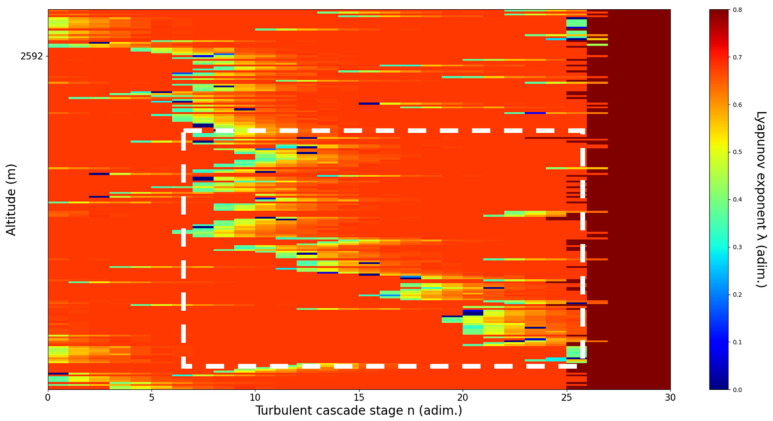
Lyapunov exponent per turbulent cascade stage colormap altitude plot, Bucharest, Romania, 13 June 2019, 07:10:00; point of interest: 2217 m.

**Figure 17:**
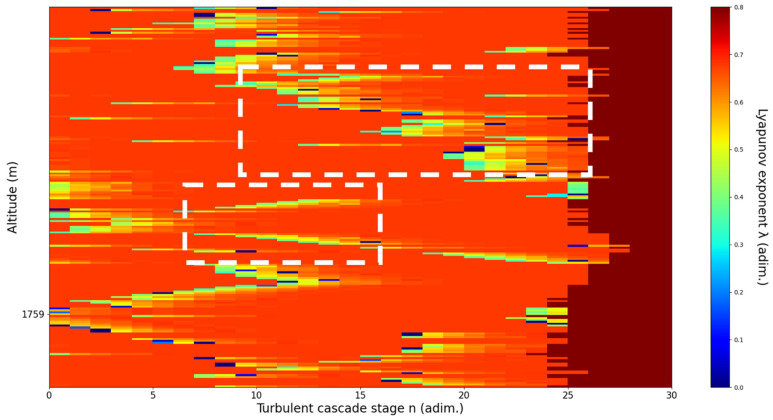
Lyapunov exponent per turbulent cascade stage colormap altitude plot, Bucharest, Romania, 13 June 2019, 07:10:00; point of interest: 1900 m.

**Figure 18:**
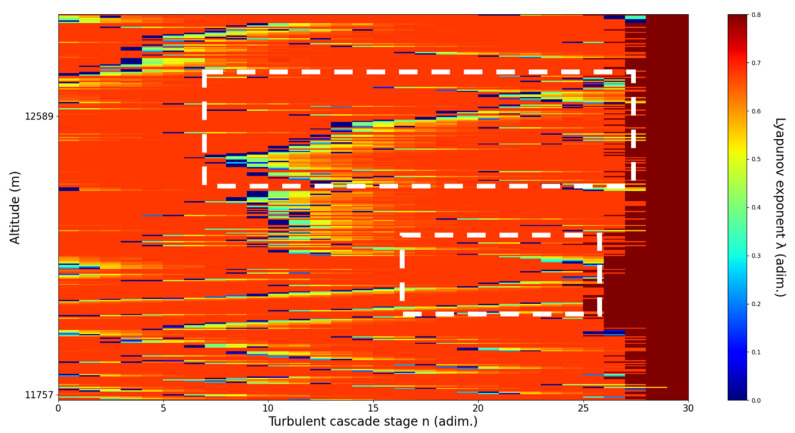
Lyapunov exponent per turbulent cascade stage colormap altitude plot, Bucharest, Romania, 13 June 2019, 07:33:00; point of interest: 12,000 m.

**Figure 19:**
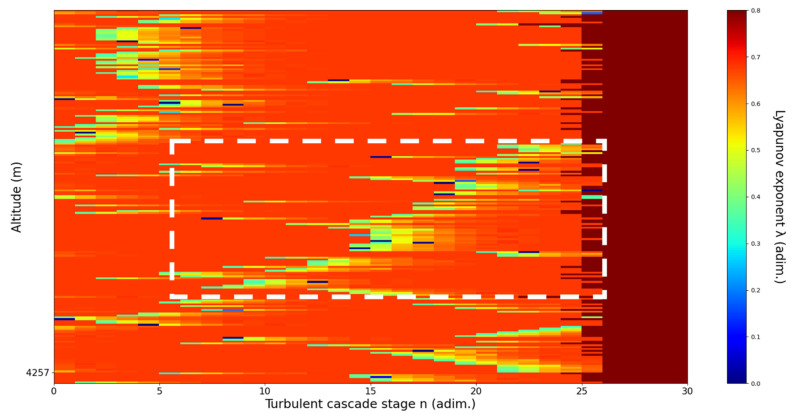
Lyapunov exponent per turbulent cascade stage colormap altitude plot, Bucharest, Romania, 13 June 2019, 07:33:00; point of interest: 4520 m.

**Figure 20:**
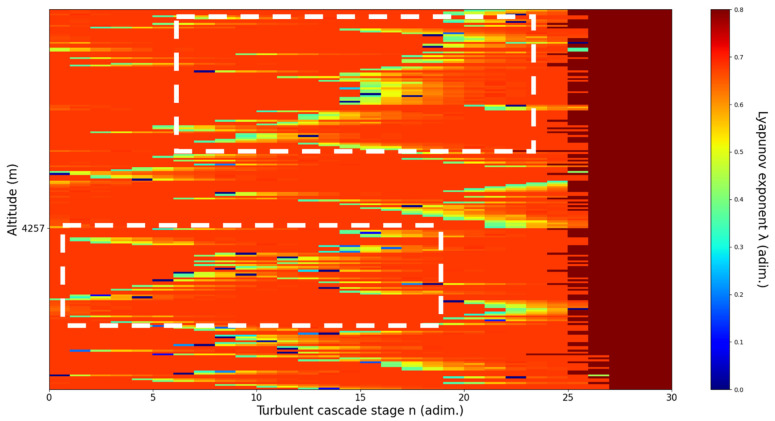
Lyapunov exponent per turbulent cascade stage colormap altitude plot, Bucharest, Romania, 13 June 2019, 07:33:00; point of interest: 4223 m.

**Figure 21:**
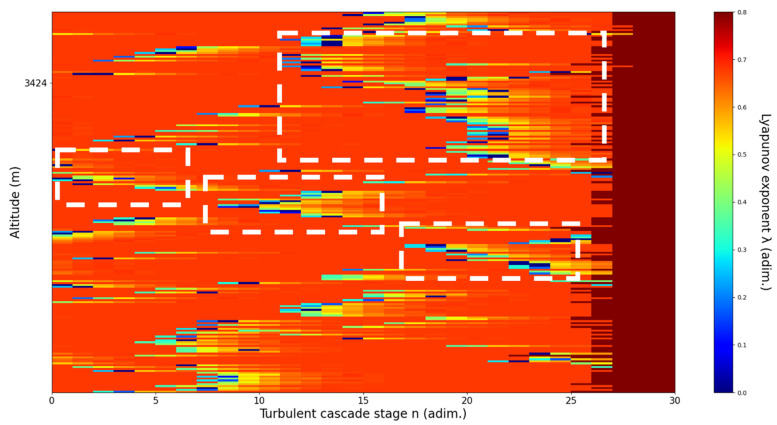
Lyapunov exponent per turbulent cascade stage colormap altitude plot, Bucharest, Romania, 13 June 2019, 07:33:00; point of interest: 3099 m.

**Figure 22:**
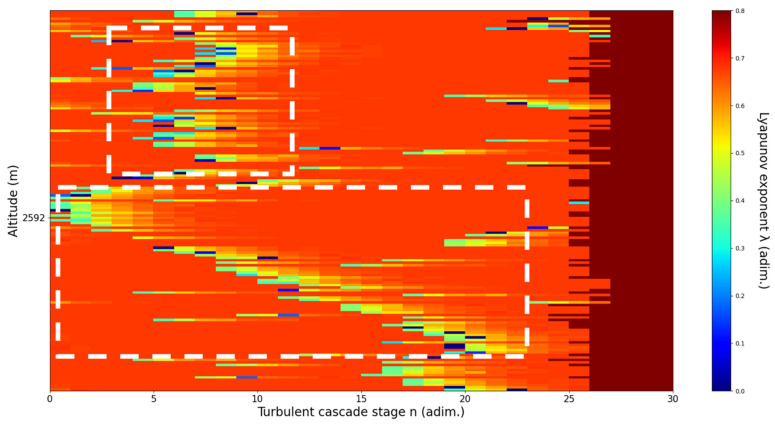
Lyapunov exponent per turbulent cascade stage colormap altitude plot, Bucharest, Romania, 13 June 2019, 07:33:00; point of interest: 2614 m.

**Figure 23:**
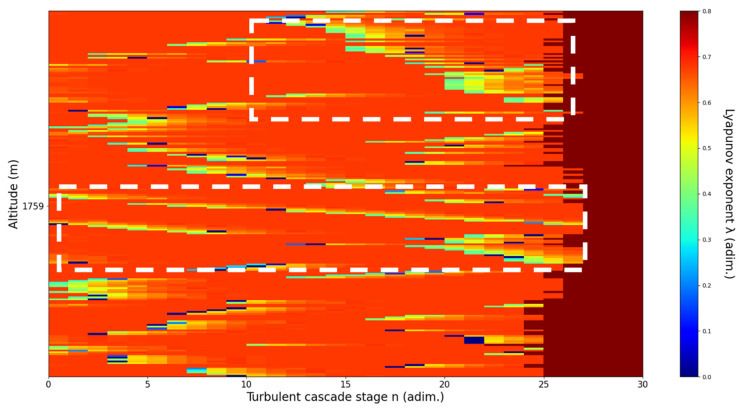
Lyapunov exponent per turbulent cascade stage colormap altitude plot, Bucharest, Romania, 13 June 2019, 07:33:00; point of interest: 1693 m.

**Figure 24:**
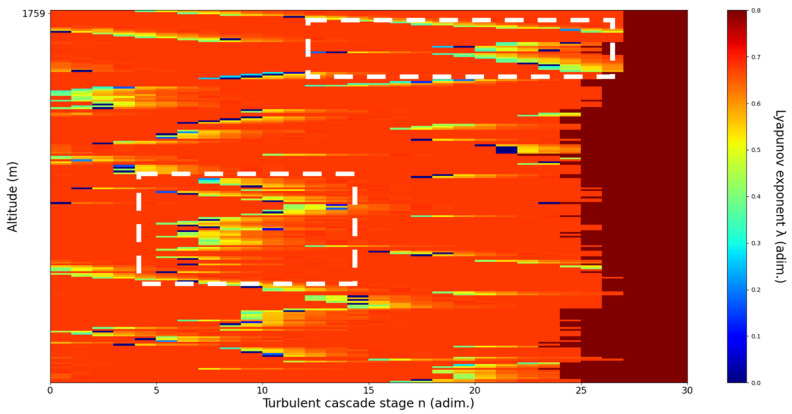
Lyapunov exponent per turbulent cascade stage colormap altitude plot, Bucharest, Romania, 13 June 2019, 07:33:00; point of interest: 1300 m.

**Figure 25:**
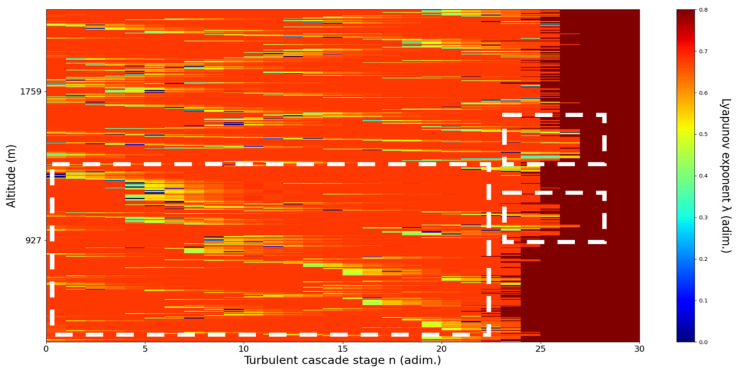
Lyapunov exponent per turbulent cascade stage colormap altitude plot, Bucharest, Romania, 13 June 2019, 07:51:00; point of interest: 1500 m.

The RCS profiles can also be used to provide a clearer picture of the atmospheric profile in comparison with the laminar analysis regarding various atmospheric formations, such as the PBL (Figure 4, Figure 5, Figure 6, Figure 7, Figure 8 and Figure 9). The laminar and turbulent environments of the profiles at the three points are analyzed in the vicinities of the following altitudes: for point A, 11,900 m, 4500 m, 4000 m, 3051 m, 2939 m, 2689 m, 2217 m and 1900 m; for point B, 12,000 m, 4520 m, 4223 m, 3099 m, 2614 m, 1693 m and 1300 m; for point C, 1500 m (Figure 10, Figure 11, Figure 12, Figure 13, Figure 14, Figure 15, Figure 16, Figure 17, Figure 18, Figure 19, Figure 20, Figure 21, Figure 22, Figure 23, Figure 24 and Figure 25). Point C presents fewer analysis altitudes because the higher altitude dynamics appear more or less like the others at similar altitudes. The presence of the large stratospheric structure throughout all sets shows that such structures could be used as an important marker for future studies in different datasets. In the following segment, the comparison between RCS profile dynamics and manifested laminar channels at certain points and altitudes is performed and discussed.

$A_{11,900m}$**: Lidar data show that the atmospheric formation is only just appearing at this point; this might reflect indeterminacy.** The analysis shows indeterminate, opposing V-shaped channels. There is some correlation between RCS data evolution and analysis (Figure 10).

$A_{4500m}$**: Lidar data show formations beginning to manifest themselves one above another; these are somewhat stable initially but quickly ascend, while the formation underneath descends.** The analysis shows a clear ascending channel above and a clear descending channel below. There is a strong correlation between RCS data evolution and analysis (Figure 11).

$A_{4000m}$**: Lidar data show the inferior part of an atmospheric structure, which is strongly fluctuating vertically.** The analysis shows V-shaped channels at chosen altitude, perhaps reflecting the indeterminate potential of the structure to either ascend or descend. There is some correlation between RCS data evolution and analysis (Figure 12).

$A_{3051m}$**: Lidar data show a thin plume about to aggressively ascend.** The analysis shows strong ascending channels starting at chosen altitude. There is a strong correlation between RCS data evolution and analysis (Figure 13).

$A_{2939m}$**: Lidar data show a thin diffuse plume close to the previous one (**112 m**vertical distance) with a descending behavior instead of an ascending one.** The analysis shows a weak descending laminar channel above the chosen altitude and a short descending laminar channel slightly above the chosen altitude, besides indeterminate horizontal channels. There is some correlation between RCS data evolution and analysis (Figure 14).

$A_{2689m}$**: Lidar data show a thin plume aggressively descending.** The analysis shows indeterminate horizontal laminar channels. There is no correlation between RCS data evolution and analysis (Figure 15).

$A_{2217m}$**: Lidar data show multiple diffuse structures descending.** The analysis shows strongly descending laminar channel. There is a strong correlation between RCS data evolution and analysis (Figure 16).

$A_{1900m}$**: Lidar data show very aggressive descent of PBL.** The analysis shows weak V-shaped channels at the altitude of the layer, and strong descending laminar channels above and below. Additionally, the analysis shows an increased number of turbulent stages near the PBL. There is a strong correlation between RCS data evolution and analysis (Figure 17).

$B_{12,000m}$**: The formation is descending, however, aggressive ascendence immediately before this point.** The analysis shows indeterminate horizontal channeling, V-shaped channels below, and strong ascending channels above. Given the fact that the analysis requires an averaging of profiles, taken from before and after the chosen points, it is possible that the analysis reflects past ascendance. There is some correlation between RCS data evolution and analysis (Figure 18).

$B_{4520m}$**: Lidar data show thin plumes fluctuating, one of them slightly ascending while another slightly descending.** The analysis shows ascending laminar channels. There is no correlation between RCS data evolution and analysis (Figure 19).

$B_{4223m}$**: Lidar data show a boundary between two relatively diffuse structures, about to descend.** The analysis shows multiple V-shaped channels below chosen altitude and ascending laminar channels above. There is a negative correlation between RCS data evolution and analysis (Figure 20).

$B_{3099m}$**: Lidar data show a thin plume aggressively descending.** The analysis shows multiple weak descending laminar channels at and above the chosen altitude, and two weak ascending laminar channels under the chosen altitude. There is some correlation between RCS data evolution and analysis (Figure 21).

$B_{2614m}$**: Lidar data show definite atmospheric structure aggressively descending.** The analysis shows a weak indeterminate horizontal laminar channel above the chosen altitude and a strong descending laminar channel under the chosen altitude. There is a strong correlation between RCS data evolution and analysis (Figure 22).

$B_{1693m}$**: Lidar data show strongly fluctuating and descending PBL.** The analysis shows several descending laminar channels at and around the chosen altitude, a strong descending laminar channel above the chosen altitude and several weak slightly ascending channels under the chosen altitude. Additionally, the analysis shows an increased number of turbulent stages near the PBL. There is some correlation between RCS data evolution and analysis (Figure 23).

$B_{1300m}$**: Lidar data show a rapidly-filling area of lower signal between two thick layers, the one above descending and the one under ascending.** The analysis shows an indeterminate area with multiple ascending and descending laminar channels; this area might function as a control, given the fact that this segment of the atmosphere neither ascends nor descends, but instead represents a progressing confluence of two layers. There is some correlation between RCS data evolution and analysis (Figure 24).

$C_{1500m}$**: Lidar data show a rapidly fluctuating and ascending yet descending very shortly after PBL.** The analysis shows ascending laminar layers above the chosen altitude and various descending laminar layers under the chosen altitude. Additionally, the analysis shows an increased number of turbulent stages near the PBL. There is some correlation between RCS data evolution and analysis (Figure 25).

Regarding the scale laminarity index analysis, segments of these profiles at PBL altitudes and in the inferior vicinity of PBL altitudes show very low values, as expected; however, relatively large peaks occur right above the PBLH in all instances (Figures 28, 31 and 34). Furthermore, such peaks are also very stable, not exhibiting fluctuations that are typical of the rest of the profile. This is further shown in the increase of the number of stages in the general laminar analysis corresponding to such areas (Figure 17, Figure 23 and Figure 25). A large-scale laminarity index can thus be produced not just by higher Lyapunov exponents at larger scales, which is indicative of active turbulence, but also simply by a greater number of stages that add up in the index calculation. It must also be mentioned that a larger number of turbulent stages also points to lower values of turbulent dissipation rate, which means that the turbulent cascades associated with the flow at that altitude are more “resilient”. In any case, below these areas of a greater number of stages, there often appear short areas of very low Lyapunov coefficients at larger scales, which might point to large-scale quasi-laminarity in the PBL (Figure 17, Figure 23 and Figure 25).

**Figure 26:**
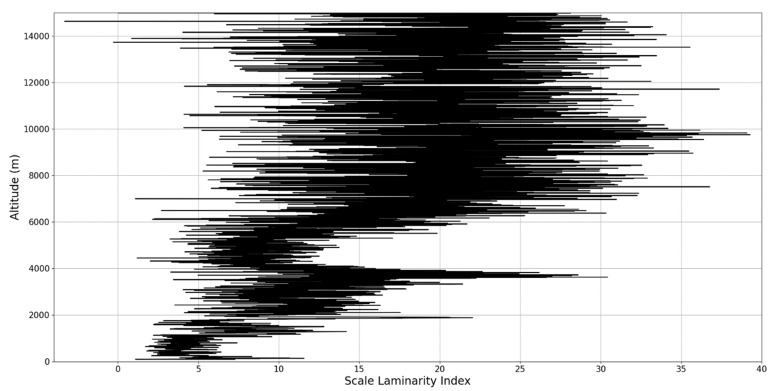
Scale laminarity index profile plot corresponding to point A.

**Figure 27:**
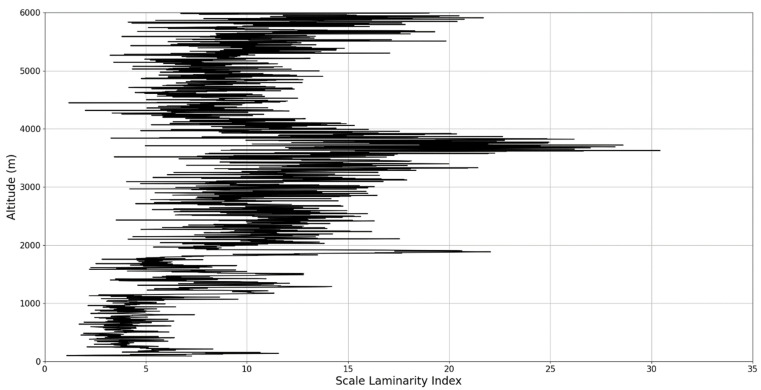
Zoomed-in scale laminarity index profile plot corresponding to point A.

**Figure 28:**
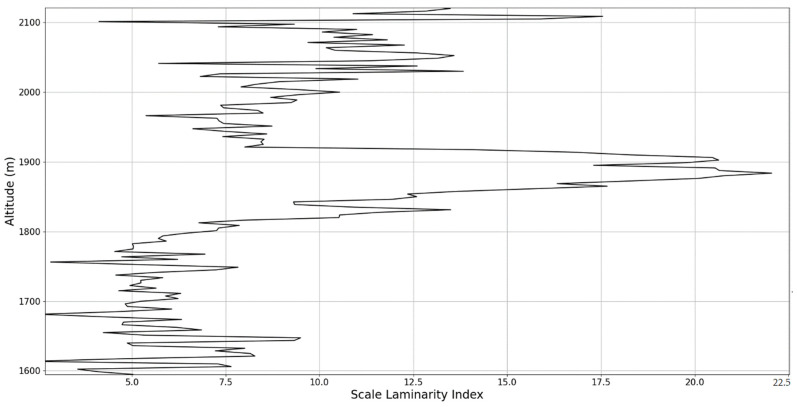
Scale laminarity index profile plot corresponding to point A in the vicinity of the PBLH.

**Figure 29:**
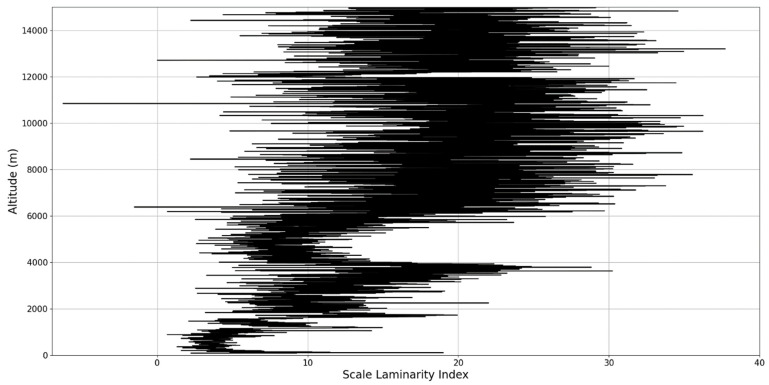
Scale laminarity index profile plot corresponding to point B.

**Figure 30:**
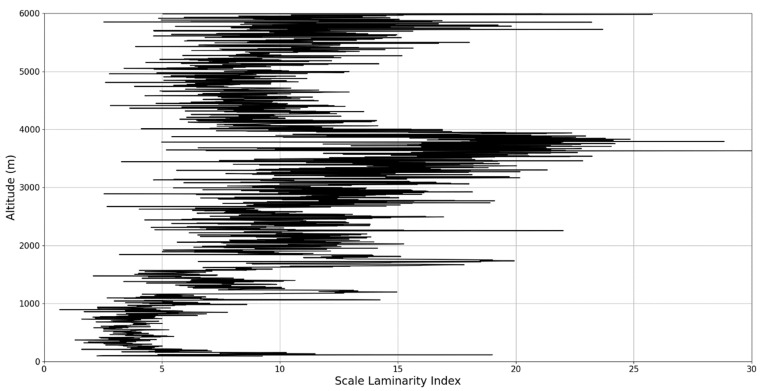
Zoomed-in scale laminarity index profile plot corresponding to point B.

**Figure 31:**
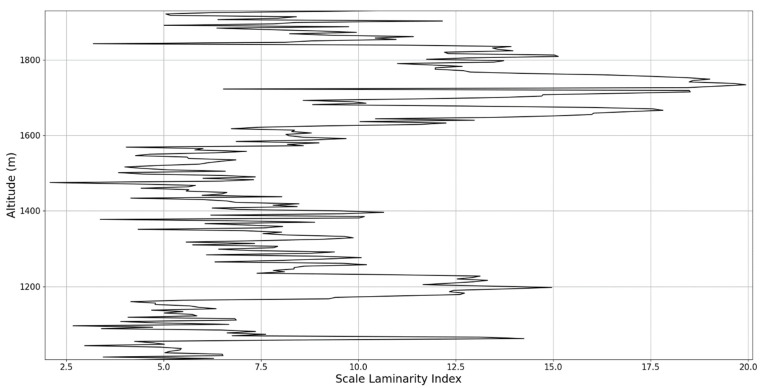
Scale laminarity index profile plot corresponding to point B in the vicinity of the PBLH.

**Figure 32:**
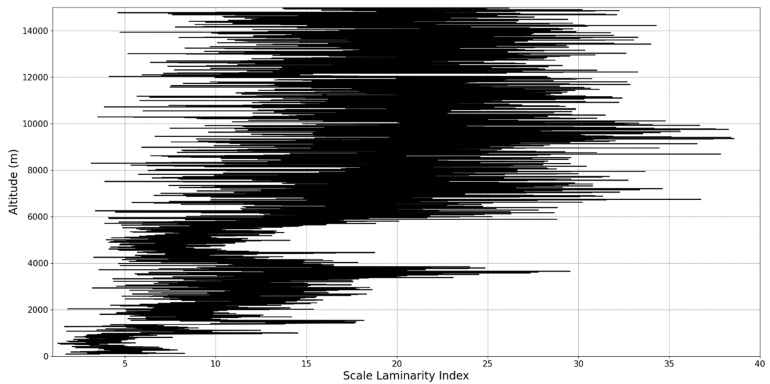
Scale laminarity index profile plot corresponding to point C.

**Figure 33:**
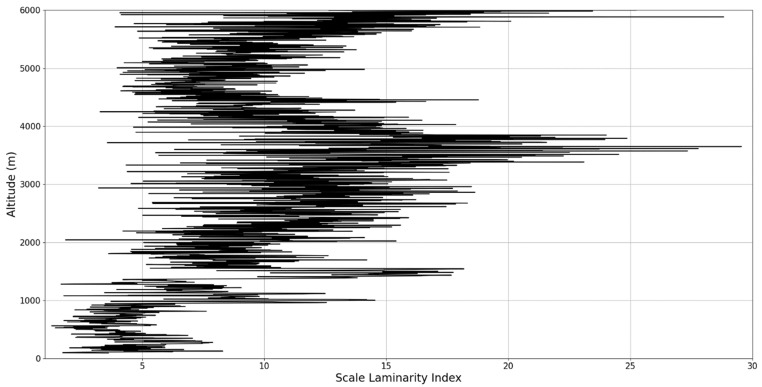
Zoomed-in scale laminarity index profile plot corresponding to point C.

**Figure 34:**
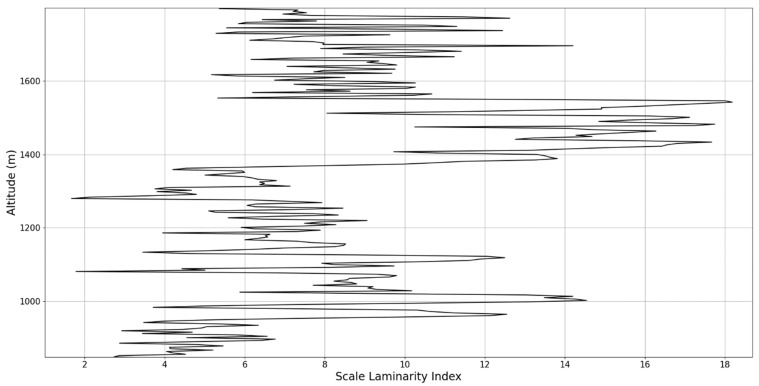
Scale laminarity index profile plot corresponding to point C in the vicinity of the PBLH.

Overall, our analysis has yielded 5 instances of strong correlation, 7 instances of correlation, 2 instances of null correlation and 1 instance of negative correlation. Furthermore, the scale laminarity index analysis shows consistent results for identifying atmospheric PBL structures (Figure 26, Figure 27, Figure 28, Figure 29, Figure 30, Figure 31, Figure 32, Figure 33 and Figure 34). These are favorable results for our theory that vertical atmospheric dynamics could be predicted by examining laminar channel trends; however multiple aspects of the comparison must be considered. First of all, the correlation has thus far been visual and arbitrary steps must be taken to quantify the differences between the RCS data evolution and the trends of the produced laminar channels and to automate this process. Second of all, the number of analyzed cases is still relatively low; both of these aspects appear as a consequence of the difficult computational and conceptual nature of the analysis itself, and this shall be addressed in the following segment. There is also the matter of employing different models and methods to quantify the directions and altitude changes of atmospheric dynamics—such a proposed method would be through the NOAA HYSPLIT model, which has been previously used successfully in concert with lidar data for vertical and temporal aerosol variation [25,26].

## 4. Tool Dissemination

Now, the study of atmospheric turbulence and laminarity intermittency and of various correlations between ascending and descending laminar channels can be fruitful, however, it is also a lengthy and difficult process. Any new theory that involves processes for which there is no set methodology, because of the novelty of the process, requires both interpretation and a certain level of speculation. However, beyond the act of interpreting this data, the sheer volume of possible laminarity data and the number of calculations necessary to obtain these data makes the experimentation stage an even more difficult task. Technically, as few as three lidar profiles are required in order to calculate a $C_{N}^{2}$ profile, however, then the software employed in this study must calculate the scales of the entirety of the turbulent energy cascade at a single given altitude out of thousands of possible altitudes in each profile. Not only that, but then each scale must yield an r which must then be used to find the behavior of the logistic map at that given r, and these values must then be associated to their respective and correct Lyapunov coefficients. Combining all this with the fact that interactive profiles must then be constructed with these data means that the production of a single instance of the figures shown in this work is a computationally intensive task, at least for a common laptop or desktop computer.

This fact prevents any ease of experimentation; furthermore, ideally, these theories should be tested on many more sets of lidar data. For these two issues, and to improve the dissemination of these ideas, the Python script used in this paper has been compiled, such that it can be used whether or not a researcher has installed the necessary Python libraries and packages. Compiling was performed using PyInstaller, first by adding Python to Windows PATH variable, and then inputting the command “pyinstaller ‘SCRIPTNAME’.py” with the flags “onefile” and “hidden-import cftime”. The dynamic link library “python38.dll” was also added in the same file as the executable. The program executes the following visible steps:

1. Opens a Windows Explorer tab which allows the user to select the desired lidar data;
2. Displays emission channel wavelengths and index associated with channel, prompting the user to select one by typing the desired index;
3. Displays the time and index associated with each lidar profile, prompting the user to select one by typing the desired index;
4. Displays “Loading…” messages until the calculations are complete;
5. Displays interactive plots.

**Figure 35:**
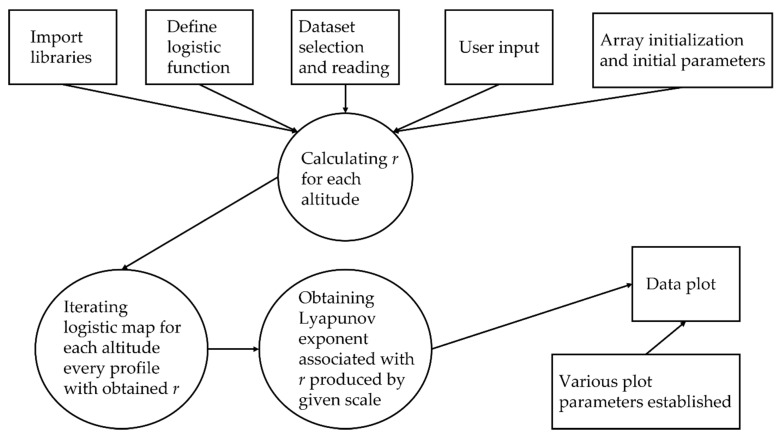
Algorithm workflow.

**Figure 36:**
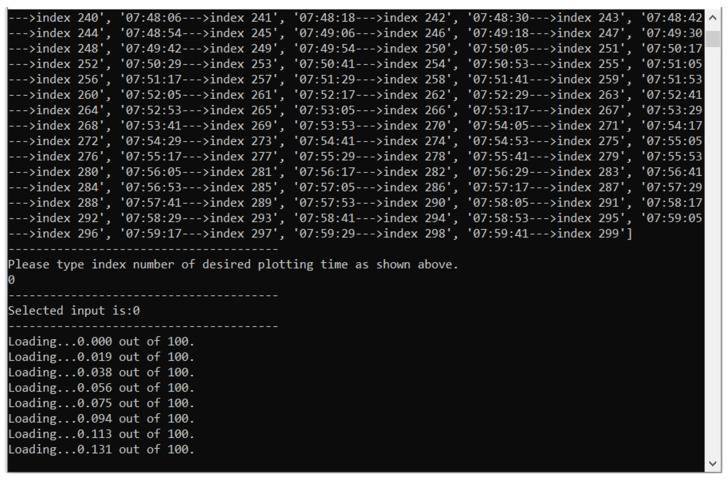
Screenshot of software.

A rough workflow and an example of the application interface are shown in Figure 35 and Figure 36.

The actual executable to be opened is titled “logistic_lidar_compile_script.exe” and it can be found in the “dist” file—obviously, the user has the possibility to create shortcuts. The input file itself has to be a NetCDF-type file, formatted by AERONET network standards; manipulating data contained in this file can be done in multiple ways, however in this case it is done through the “netCDF4” library for Python. The functionality of the produced plots is based on the library “matplotlib”, as such their size and margins can be modified, they can be zoomed-in or out in various areas, and they can be saved in multiple formats. The program produces four plots: an RCS time series of the whole dataset, a single RCS profile of the chosen index, the laminar channel analysis plot, and the scale laminarity index plot, which is to say it produces the same plots that were previously analyzed.

Multiple useful functionalities can be found in the plots themselves; in the upper-left corners of the plot windows, the buttons “Reset original view”, “Back to previous view”, “Forward to next view”, “Pan”, “Zoom”, “Configure subplots”, “Figure options” (or “Edit axes, curve and image parameters”) and “Save”. Many of these are self-explanatory, however, minor instructions are required for the sixth and seventh buttons. The “Configure subplots” button allows for instant modification of plot size or borders, or for establishing a “Tight layout” of the plot; the “Export values” button allows for easy copying of plotting size parameters. Meanwhile, the “Figure options” functionality allows the user to modify plotting axes limits and scaling, from linear to logarithmic, and even to add or remove labels; in the “Images, etc.” tab it is also possible to instantly modify the colormap of the plot wherever possible, and to modify the minimum and maximum limits of the chosen colormap, thus fundamentally modifying the aspect of the plot and potentially highlighting important features. Of course, the authors of this work encourage other researchers to test these theories through multiple plot analysis and to communicate any error or desired functionality–the software used and discussed in this chapter can be found and downloaded through the link provided in the Supplementary Materials section.

## 5. Conclusions

In this work, a more thorough practical examination of our previous research has been performed, with positive results that point to theoretical validity. After a compressed recapitulation of the required theory, and an explanation of the used theoretical procedures, plots were created that point not only to the existence of atmospheric laminar channels but also to the fact that such channels may dictate the vertical dynamics of macroscopic atmospheric structures, such as aerosol plumes or even the PBL itself. In comparison to RCS data evolution, laminar channel data have been found to mostly yield a positive correlation. Furthermore, the scale laminarity index analysis has proven to be helpful in identifying the PBLH, exhibiting clear and coherent peaks between the PBL and the superior segment of the atmosphere. Finally, acknowledging the difficulty of fully verifying such theories using large quantities of lidar data, software used to construct such plots is disseminated by compiling the Python code used to perform it. This executable and its associated resources are then distributed, and any researchers with access to access to lidar data of the type used in this study are invited to employ these tools in new studies. Further studies will need to be focused on finding ways to quantify the differences and similarities between laminar analysis and vertical atmospheric dynamics through correlation and to do so with much larger quantities of data.

## Data Availability

The data presented in this study are available on request from the corresponding author.

## Supplementary Materials

The following are available online at https://drive.google.com/file/d/1nvnJfr2KM9QoTmeCLrpO2Ac3G2kxlNgl/view?usp=sharing: tool used to create plots shown in this paper, including libraries and executable (accessed on 4 November 2021).

## Nomenclature

ε	scale resolution
$L_{0}$	initial length scale
$l_{d}$	dissipation length scale
β	scale ratio
Re.	Reynolds number
k	velocity field wavenumber
τ	time step parameter
r	logistic map control parameter
$u_{d}$	dissipation velocity scale
$\lambda_{n}$	Lyapunov indices
$S_{\lambda }$	scale laminarity index
$C_{N}^{2\;}$	structure coefficient of the refraction index profile
$\;\sigma_{I}^{2}$	scintillation of the intensity of the backscattered range-corrected lidar signal
I	intensity of the backscattered range-corrected lidar signal
RCS	(Range Corrected Signal)
PBL	(Planetary Boundary Layer)
PyInstaller	tool for compiling Python applications into stand-alone executables
NetCDF	(Network Common Data Form)
